# An Unusual Occurrence of Multiple Dental Anomalies in a Single Nonsyndromic Patient: A Case Report

**DOI:** 10.1155/2012/426091

**Published:** 2012-06-25

**Authors:** N. B. Nagaveni

**Affiliations:** Department of Pedodotnics and Preventive Dentistry, Hitkarini Dental College and Hospital, Madhya Pradesh, Jabalpur 482005, India

## Abstract

Dental anomalies are the formative defects caused by genetic disturbances or environmental factors during tooth morphogenesis. Simultaneous occurrence of various multiple anomalies has been reported previously, particularly in cases of chromosomal abnormalities that often manifest with multisystem involvement. Very few cases of multiple anomalies have been documented in patients with no known generalized abnormalities. The present paper shows an unusual occurrence of a combination of dental anomalies like mandibular canine transmigration, taurodontism in permanent mandibular molars, congenital agenesis of 14 numbers of permanent teeth excluding third molars, canine impaction, primary molars with pyramidal roots, midline diastema and generalized microdontia in an apparently normal 13-year-old Indian girl.

## 1. Introduction

Odontogenic anomalies are the formative defects caused by genetic disturbances or environmental factors during tooth morphogenesis. Occurrence of multiple anomalies in individuals or families, without evidence of other systemic manifestations or syndromes have rarely been reported [1–3]. Desai et al. [1] described concomitant occurrence of idiopathic generalized short root anomaly and microdontia, taurodontism of posterior teeth, obliterated pulp chambers, infected cyst, and multiple dens invaginatus. Dash et al. [2] presented a case of talon cusp affecting the mandibular central incisor and maxillary lateral incisor, an inverted impacted migrating mandibular second premolar, and hypodontia. Recently Suprabha et al. [3] showed presence of multiple dens invaginatus, generalized enamel hypoplasia, generalized microdontia, root resorption and multiple periapical lesions, and supernumerary teeth.

Preeruptive, intraosseous migration of a tooth across the midline is termed “transmigration” [4–6]. It is evident from the literature that it occurs more frequently in females than in males. This phenomenon usually affects the permanent canine, premolar, or lateral incisors with mandibular canine being the most frequently involved tooth [4–6]. The reported patient age at presentation of the transmigrant canine varies from 8 years to 69 years and may occur either unilaterally or bilaterally [4]. The causes underlying this condition remain unclear although a number of theories have been proposed. It is generally accepted that the impacted tooth follows the trajectory of least resistance. The tooth moves in the direction of the crown, and the mesial inclination of the follicle makes it possible to migrate toward the contralateral side. However, traumatic factors, heredity, the long eruption path of canine tooth germs, premature loss of primary teeth, filling of this space by an adjacent tooth, disharmony of tooth-size, unfavorable alveolar arch length, and over length of crowns can be the causative factors [4, 6].

Taurodontism is a developmental disturbance of a tooth that lacks constriction at the level of the cementoenamel junction and is characterized by vertically elongated pulp chambers, apical displacement of the pulpal floor, and bifurcation or trifurcation of the roots [7]. The prevalence of taurodontism is reported to range from 2.5% to 11.3% of the human population [8–11]. This condition has been reported in both primary and permanent teeth, with a prevalence of approximately <1% and 3% to 35%, respectively, depending on the population studied and diagnostic criteria used [8–11]. In the permanent dentition, the second and third molars are more commonly affected than the first molars. This trait can be seen in a single tooth, or in several teeth in the same quadrant, and can be unilateral or bilateral. The etiology of taurodontism is unclear. The possible causes of taurodontism have been enumerated by Mangion [12] as follows: a specialized or retrograde character, a primitive pattern, a mendelian recessive trait, an atavistic feature, and a mutation resulting from odontoblastic deficiency during dentinogenesis of the roots.

Oligodontia is a developmental dental anomaly refers to congenital missing of more than six teeth excluding third molars [13]. Its prevalence varies from 0.08% to 0.16% [14]. The difference in frequency of oligodontia between males and females is not significant, nor is the difference in distribution of missing teeth in maxilla/mandible and right/left sides [14]. However, combining data from various studies shows a higher frequency in females.

Transmigration, taurodontism, oligodontia, microdontia, and canine impaction individually have been previously reported [4–14]. The combined occurrence of these anomalies, however, has not been reported till date. The present case report describes an Indian girl of 13 years of age in whom the above-mentioned anomalies like transmigration, taurodontism, oligodontia, microdontia, impaction and pyramidal primary molar roots were present. Occurrence of these number of anomalies in a single patient without known any other abnormalities or syndromes is certainly a rare case and possibly unique.

## 2. Case Report

**Figure 1:**
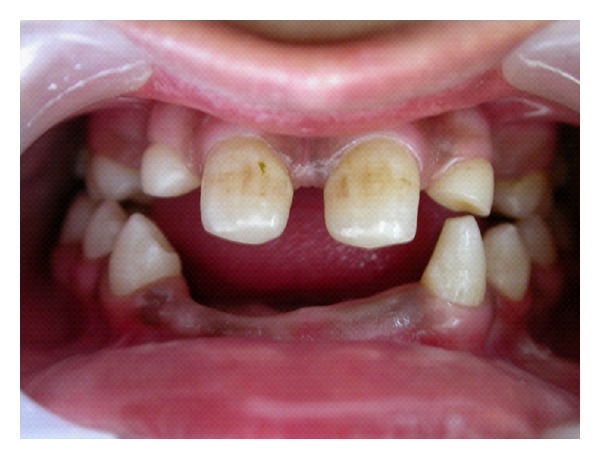
Missing lower anterior teeth.

**Figure 2:**
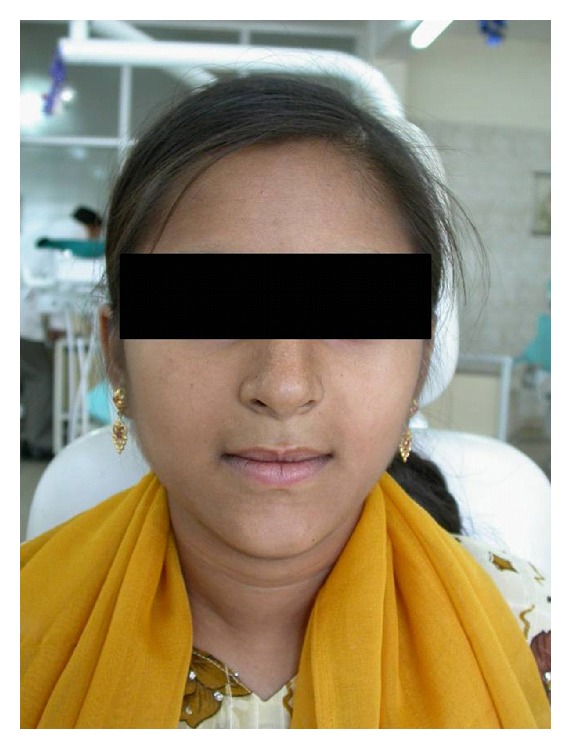
Facial appearance of patient.

A 13-year-old female patient reported complaining of missing teeth in the lower anterior region. (Figure 1). Patient mother gave a history of presence of milk teeth in the lower arch, but, after their exfoliation, permanent teeth did not erupt. There was no history of any infection or trauma to the anterior region. Patient was healthy with no relevant medical and family history and was born to nonconsanguineous parents. She was normal in her facial appearance and did not show any physical or skeletal abnormality (Figure 2). Radiological examinations of the clavicles, vertebral skeleton, skull, and chest were found to be normal. Ophthalmological and neurological examination of the patient revealed no pathological symptoms and showed no signs of mental retardation. Hematological and biochemical findings were found within normal limits.

**Figure 3:**
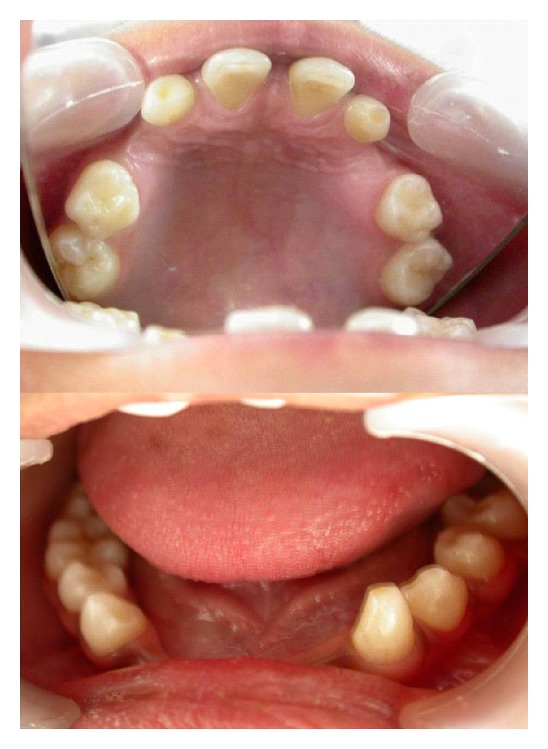
Upper and lower arch showing missing teeth.

**Figure 4:**
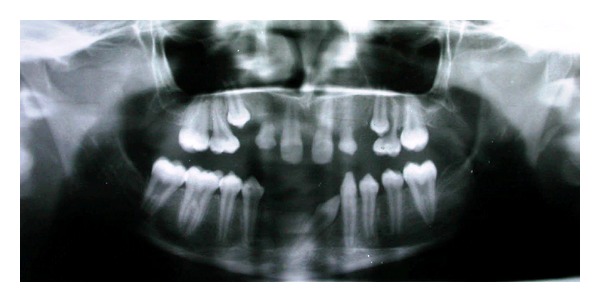
Orthopantomograph showing multiple dental anomalies.

Intraoral examination revealed midline diastema and presence of permanent teeth like 11, 16, 21, and 26 in the maxillary arch, 34, 35, 36, 44, 45, 46, and, 47 in the mandibular arch. Primary teeth like 53, 55, 63, 65, and 73 were retained (Figure 3). The remaining permanent teeth were missing clinically. On radiographic examination, congenital absence of 12, 13, 15, 17, 22, 23, 24, 27, 31, 32, 33, 37, 41, and 42 (14 teeth) was confirmed (Figure 4). All third molar teeth buds were also missing. Permanent mandibular right canine was impacted mesioangularly with crown transmigrated to contralateral side crossing the midline and was rotated. Other anomalies also noticed on the radiograph like, taurodontism in mandibular right first and second molars and left first molar, pyramidal or conical shaped, fused roots in maxillary both primary and permanent molars and generalized microdontia.

In view of the oligodontia of permanent teeth, a detailed examination was done to rule out syndromes or systemic diseases associated with oligodontia and found negative. Finally, a diagnosis of nonsyndromic occurrence of multiple dental anomalies was made. As patient was mainly concerned with missing lower anterior teeth, removable partial denture fabrication was done to restore esthetics and masticatory function.

## 3. Discussion

The combination of mandibular canine transmigration, a relatively rare developmental dental anomaly, together with its impaction and complete agenesis of 14 permanent teeth excluding third molars (oligodontia), taurodontism of molars, canine impaction, microdontia and pyramidal or fused, conical rooted maxillary molars makes this case an interesting one, particularly since the condition is not associated with any known systemic diseases or developmental syndrome.

Mupparapu [15], in 2002, presented nine cases of transmigration and proposed a classification system. The author classified these teeth into five types based on their migratory pattern and their position in the jaw. The classification can be summarized as the following.

Type 1: canine impacted mesioangularly across the midline, labial, or lingual to the anterior teeth with the crown portion of the tooth crossing the midline.
Type 2: canine horizontally impacted near the inferior border the mandible inferior to the apices of the incisors.
Type 3: canine erupting on the contralateral side
Type 4: canine horizontally impacted near the inferior border of the mandible below the apices of posterior teeth on the opposite side
Type 5: canine positioned vertically in the midline with the long axis of the tooth crossing the midline.

Javid [16] suggested that an impacted tooth that has crossed the midline more than half of its length should be considered as transmigrated. On the other hand, it is also stated that the more important consideration should be the tooth crossing the midline, not the distance of the migration [17]. Mupparappu's [15] nine patient series also showed teeth with only the crown crossing the midline. In addition, the stage of transmigration will depend on the time of diagnosis. In light of these opinions, the present case was considered as a true transmigration, fitting the criteria for Type 1, as it was “positioned mesioangularlyacross the midline within the mandible and the crown of the tooth crossing the midline.”

Literature search revealed transmigration of teeth associated with other pathological entities like follicular cysts, odontoma, and hyperdontia [4, 17–19]. To the best of author's knowledge, occurrence of oligodontia, taurodontism, and microdontia in association with canine transmigration is not documented so far thereby making this case as an unusual entity.

Oligodontia occurs as an isolated trait or as a part of other systemic or syndromic abnormalities. Isolated oligodontia is inherited as an autosomal dominant form with reduced penetrance. Tsai et al. [20] reported a case of oligodontia in 6-year-old female patient with congenital absence of 16 permanent teeth. Akkya et al. [21], in their case of 16-year-old patient, found missing of 6 permanent teeth. Rasmussen [22] presented nonsyndromic 9 cases with absence of 14–24 teeth. The present report showed congenital agenesis of 14 permanent teeth excluding third molars.

Oligodontia is seen in association with a tendency toward delayed tooth formation and reduced size of the teeth [14]. It is also proposed that oligodontia is more likely to be associated with other developmental changes such as taurodontism. In a Dutch study [23] of patients with oligodontia, 28.9% showed taurodontism of one or two mandibular first molars, while 9.9% of the control subjects had taurodontism. This can be attributed to the fact that a number of genes expressed in the early stages of tooth development, namely, MSX1, PAX9, and AXIN2 have been linked to oligodontia, whereas genes expressed later during root morphogenesis such as ALPL have been identified in taurodontism. These genes, which are expressed at 2 distinctly different points in time during the entire tooth formation process, are likely to provide the link between oligodontia and taurodontism [24].

The third important anomaly reported in this case is the taurodontism affecting the permanent mandibular molars. Taurodontism has been reported either as an isolated anomaly or as a feature of multiple-system malformation syndromes such as ectodermal dysplasia, Down syndrome, Klinefelter syndrome, tricho-dento-osseous syndrome, and X-linked hypophosphatemic rickets [25]. Taurodontism has also been found in association with various dental anomalies, including amelogenesis imperfecta and hypodontia. Taurodontism, short roots, and external resorption have been described in patients with small head and short stature. However, it occurs in 2.5% to 3.5% of chromosomally normal Caucasians. Shaw [26] classified this condition as hypo-, meso- or hypertaurodontism, based on the degree of apical displacement of the pulp chamber floor. Hypotaurodontism is the least pronounced form, in which the pulp chamber is enlarged, mesotaurodontism is the moderate form, in which the roots are divided only at the middle third, and hypertaurodontism is the most severe form, in which bifurcation or trifurcation occurs near the root apices. According to this classification, the taurodontic mandibular molars of this case were classified as mesotaurodonts.

In the presented case, anomalies occurred together with an impacted canine transposed to central incisor position which was congenitally missing. It is difficult to explain why this canine became completely transposed and impacted with crown migrated to cross the midline. Due to congenital absence of 41 and 42, the tooth germ of 43 had probably drifted mesially and located in place of central incisor. Later this had crossed the midline due to existing space created by congenital agenesis of 31 and 32. This is an extreme case of ectopic eruption wherein, a tooth erupts in a position normally occupied by another tooth and may be termed as transposition-transmigration. But in this case the tooth is not erupted and cause behind this is not understood. This may be attributed to local factors, thick fibromatous tissue, dense bone, soft tissues, or genetic factors.

Other dental abnormalities found in this case were generalized microdontia, midline diastema, and a marked anomaly involving the roots of maxillary both permanent and primary molars, that is, fused, conical- or pyramidal-shaped roots.

The combination of dental anomalies found in this case is probably unique and differs from previously published cases. Patients suffering from such combination of multiple anomalies need to be diagnosed early and require multidisciplinary management.
